# Vision Threatening Raised Intracranial Pressure Associated with Recreational Nitrous Oxide Use

**DOI:** 10.1080/01658107.2023.2301359

**Published:** 2024-01-26

**Authors:** Aimee Goel, Pavan S. Khatkar, Jenny L. Hepschke, Athanasios Zisakis, Susan P. Mollan

**Affiliations:** a Department of Neurosurgery, University Hospitals Birmingham, Birmingham, UK; b Medical School, Imperial College London, London, UK; c Birmingham Neuro-Ophthalmology, University Hospitals Birmingham, Birmingham, UK; d Translational Brain Science, Institute of Metabolism and Systems Research, University of Birmingham, Birmingham, UK

**Keywords:** Chronic abuse, illicit drug, laughing gas, nitrous oxide, raised intracranial pressure, recreational use, papilloedema

## Abstract

Nitrous oxide is used as an anaesthetic and analgesic agent in the medical setting and is known to cause raised intracranial pressure. The use of nitrous oxide recreationally for the drug’s euphoric and relaxant properties has been linked to multiple neurological and psychiatric sequelae including neuropathy, myelopathy, and psychosis. We describe a case of a young person who declared heavy nitrous oxide use resulting in vision-threatening papilloedema secondary to raised intracranial pressure. He underwent emergency lumbar drainage alongside high-dose acetazolamide and parenteral vitamin B_12_ injections. To our knowledge, there have yet to be other reports of cases where heavy nitrous oxide use has caused secondary pseudotumor cerebri syndrome.

## Introduction

Nitrous oxide is commonly used in the medical and dental setting as an inhalational anaesthetic agent. However, it was used recreationally for public entertainment and in British high society parties as early as the 18^th^ century, long before its medical potential was recognised.^1^ There has been a recent resurgence of recreational use of the gas. In the United Kingdom, it is the second most prevalent drug of abuse after cannabis.^2^ Apart from its haematological and cardiorespiratory risks, its neurological manifestations in the form of subacute combined degeneration of the cord and motor and sensory neuropathies are most well known. The commonest presenting neurological issues in patients with chronic nitrous oxide use are sensory ataxia and abnormal vibration and proprioception, with posterior and posterolateral column dysfunction.^3^ There have been no reports of visual loss in patients with recreational nitrous oxide use in the current English literature. We describe a case of vision-threatening papilloedema in a young person with a background of chronic, high dose recreational nitrous oxide use.

## Case report

A right-handed male student living with obesity who was in his early twenties presented 1-week following a thunderclap headache. He reported to be halfway through a nitrous oxide canister when he was suddenly affected by an intense severe headache, coming on instantly from zero to maximal pain. At the time, he was not exerting himself other than inhaling a bag of nitrous oxide. He reported that his vision became blurred and he saw non-coloured flashing lights in both eyes. He was a heavy recreational user of nitrous oxide, using balloon delivery with an estimated 0.5 litre canister daily in the preceding 4 months. He was otherwise well with no prior personal or family history of migraine and no significant past medical or family history.

**Figure 1.**
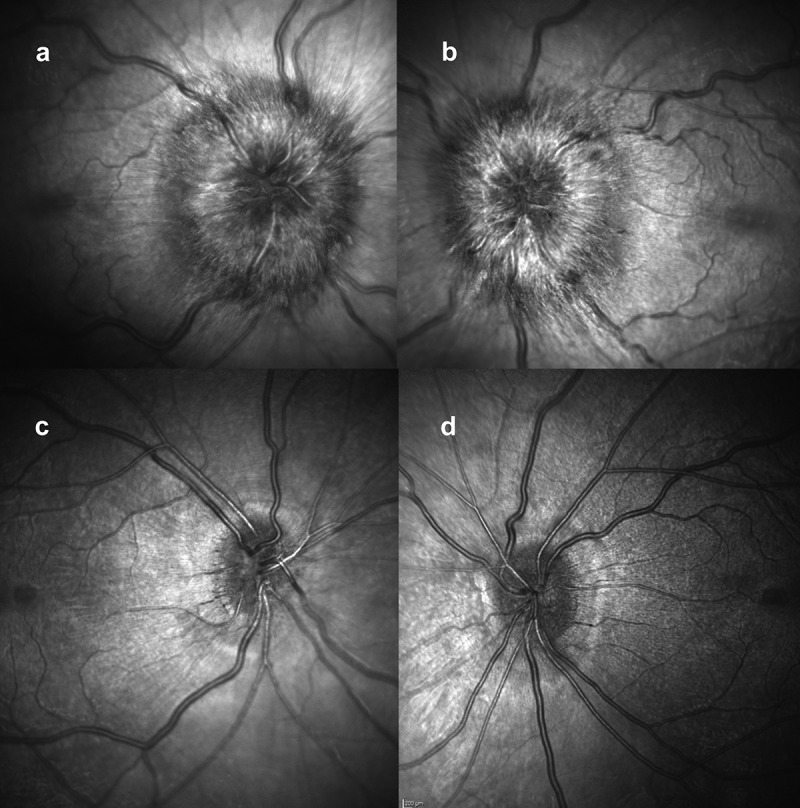
Optical coherence tomography (OCT) infrared images of the optic nerves at presentation: (a) right eye; (b) left eye, both showing papilloedema. OCT infra-red images at 2 months, following treatment: (c) right eye; (d) left eye, both without papilloedema.

**Figure 2.**
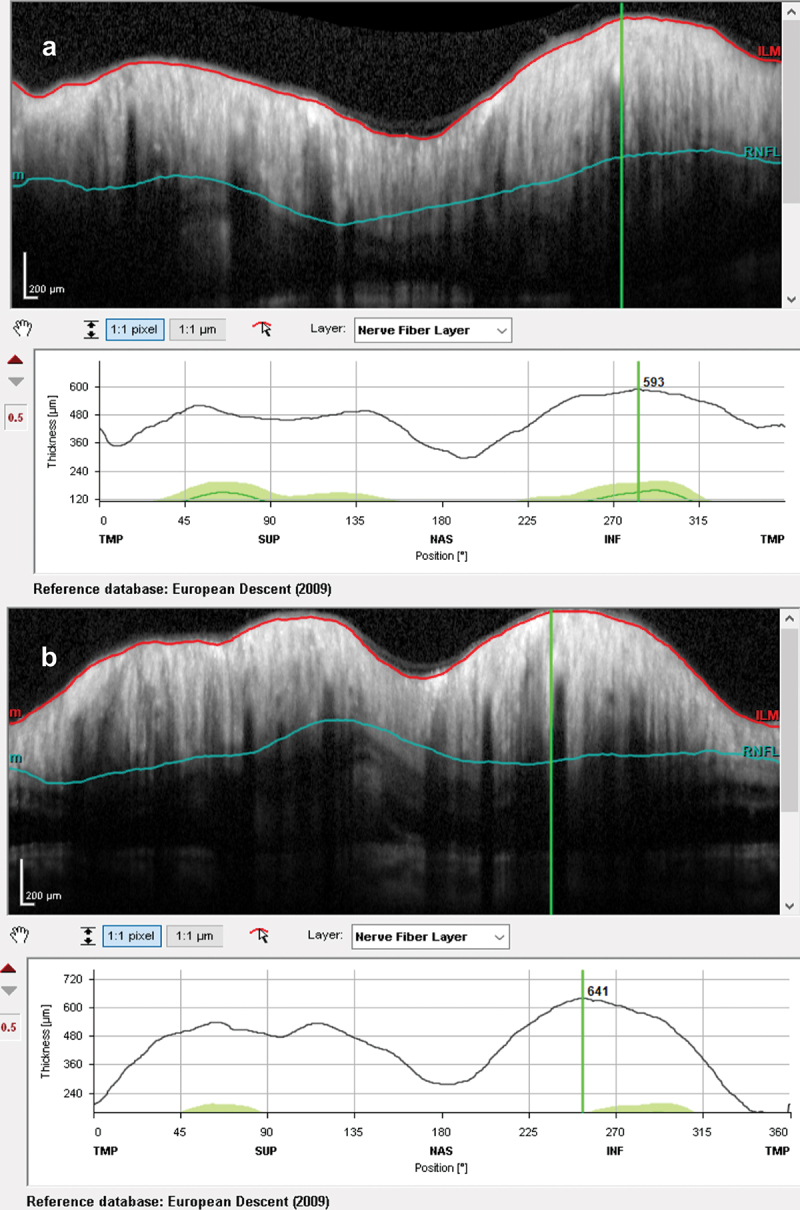
Optical coherence tomography retinal nerve fibre layer thickness (RNFL) at presentation showing increased thicknesses of the RNFL: (a) right eye and (b) left eye.

**Figure 3.**
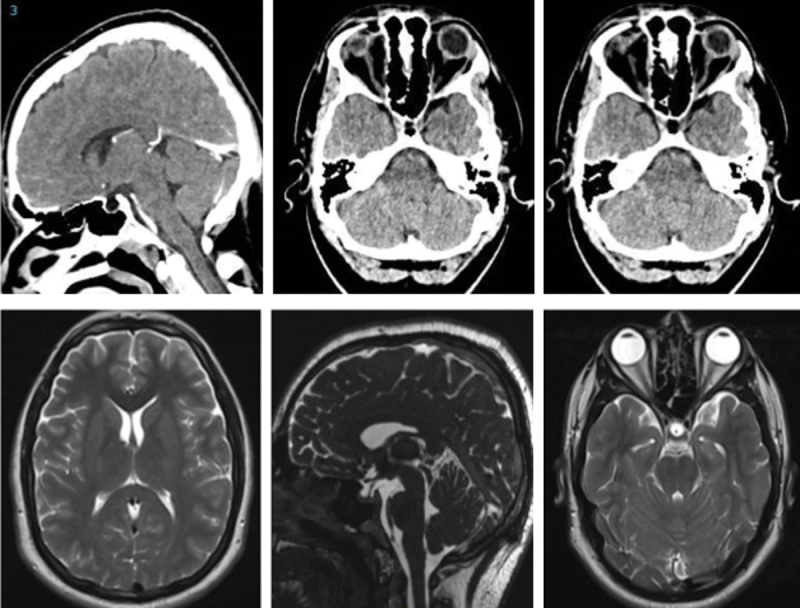
Computed tomography scans (upper row) and magnetic resonance imaging (lower row) showing a partially empty sella turcica and small ventricles, suggestive of intracranial hypertension.

At presentation, he had a moderately severe, constant, diffuse headache with photophobia and phonophobia. On examination, there were no focal neurological deficits. He had a body mass index of 32 kg/m^2^. He had reduced visual acuity of 6/12 in his right eye and 6/18 in his left eye with a left relative afferent pupillary defect. Colour vision using Ishihara plates was normal in each eye. The visual fields to confrontation showed bilateral enlarged blind spots and a left inferior nasal step. His intraocular pressures were: right eye 13 mmHg and left eye 15 mmHg. Dilated slit-lamp examination revealed symmetrical Frisén grade 5 papilloedema with haemorrhages and cotton wool spots, suggestive of mixed compressive and ischaemic optic neuropathy. His optical coherence tomography (OCT) images quantitatively revealed the extent of the papilloedema, with global peripapillary retinal nerve fibre layer (RNFL) thicknesses of 466 µm in the right eye and 437 µm in the left (Figures 1 and 2). Computed tomography (CT) scanning and magnetic resonance imaging (MRI) of the head showed a partially empty sella turcica, distortion of the optic nerves and narrowing of the transverse sinuses; features suggestive of intracranial hypertension (Figure 3). CT venography did not show a venous sinus thrombosis. MRI of the whole spine (which included the cervical, thoracic, and lumbar regions) was unremarkable. Lumbar puncture opening pressure was 51 cm of cerebrospinal fluid (CSF). CSF analysis was unremarkable. Serum inflammatory markers, haemoglobin, coagulation profile, and vitamin B_12_ levels were normal.

He underwent emergency lumbar drainage and was started on an escalating dose of acetazolamide.^4^ He was also prescribed parenteral vitamin B_12_ injections for suspected nitrous oxide-induced functional vitamin B_12_ deficiency (the initial blood sample had been insufficient to confirm vitamin B_12_ deficiency). Subsequent biochemistry tests for this were normal: the serum homocysteine level was 13 µmol/L (laboratory reference <18 µmol/L); methylmalonic acid level was requested, however the laboratory reported their test was not sufficiently sensitive for the investigation of vitamin B_12_ deficiency. After 2 days, the drain was removed against medical advice. Following discussions regarding treatment options for continued sight-threatening disease, such as a permanent CSF shunt or optic nerve sheath fenestration, the drain was re-sited for a further 5 days.

**Figure 4.**
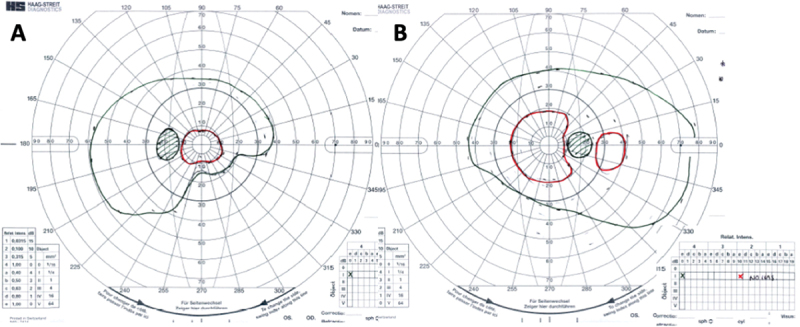
Goldmann visual fields in the (a) Left eye and (b) Right eye at 2 months, following treatment.

**Figure 5.**
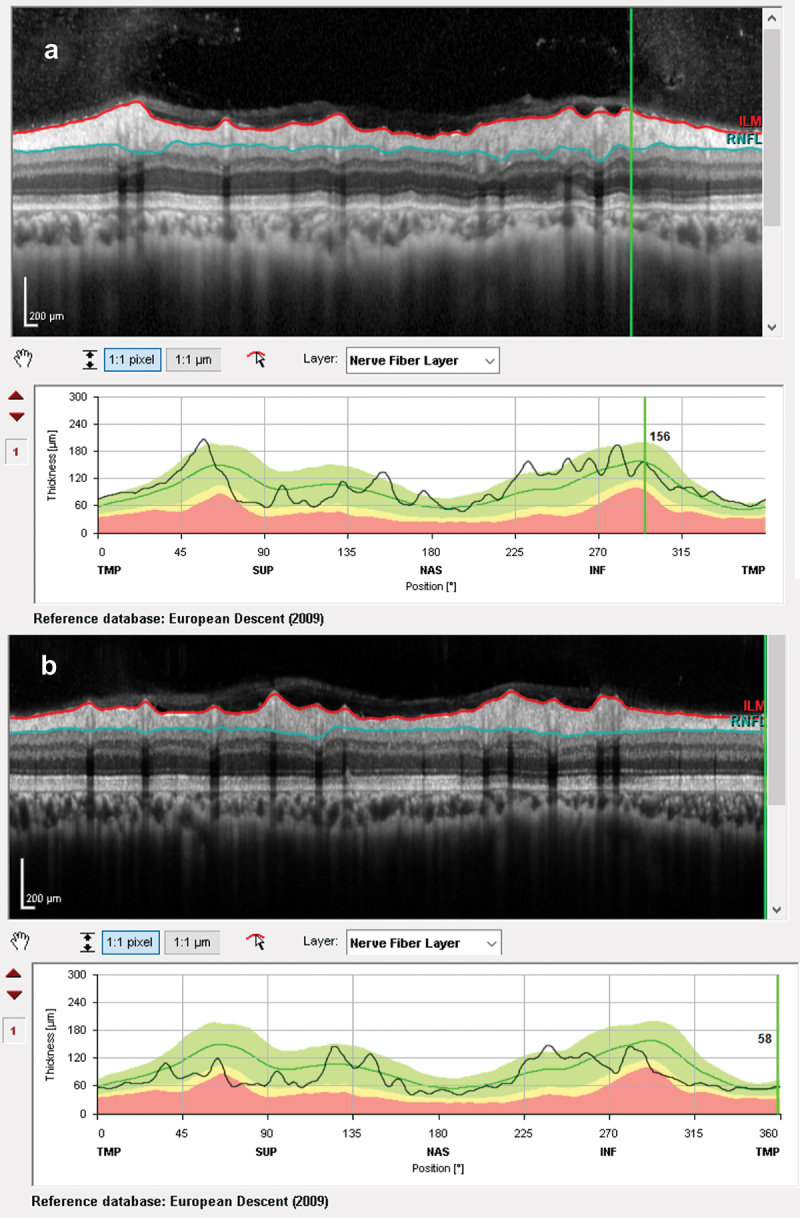
Optical coherence tomography retinal nerve fibre layer thicknesses at 2 months, following treatment: (a) right eye and (b) left eye.

**Figure 6.**
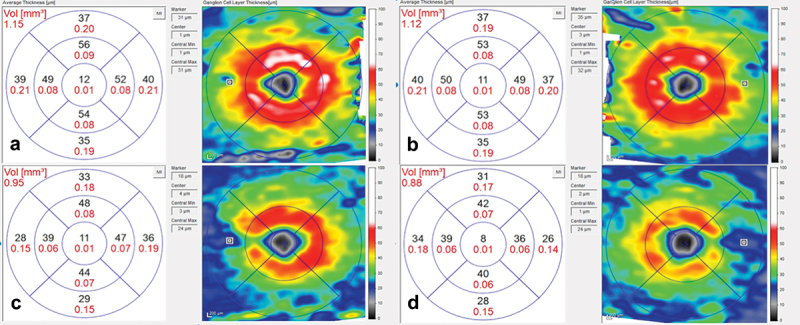
Optical coherence tomography macular ganglion cell analysis: (a) right eye at presentation; (b) left eye at presentation; (c) right eye at 2 months, following treatment and; (d) left eye at 2 months, following treatment.

Over the course of his 2-week admission, the papilloedema settled and his vision and headaches improved. At discharge, his visual acuity was 6/5 in each eye. His relative afferent pupillary defect had resolved, and his visual field had subjectively improved (Figure 4). He was advised to abstain from nitrous oxide use and reduce the acetazolamide from 2 g in a daily divided dose to stopping over the next 2 months. At 2 months, the papilloedema had resolved with global peripapillary RNFL thicknesses of 102 µm in the right eye and 81 µm in the left eye (Figures 1 and 5). His macular ganglion cell layer at 2 months was abnormal with specific loss in the temporal macular regions, worse in the left eye (Figure 6).

## Discussion

The recreational use of nitrous oxide, especially in young adults, has been rising in recent years. In the short term, it results in brief dissociative, euphoric and hallucinogenic effects and sedation. These effects are thought to be mediated through N-methyl-D-aspartate (NMDA) antagonism, decreasing excitatory neurotransmission by non-competitive glutamate inhibition.^5^ Nitrous oxide is also a partial mu, kappa, and delta opioid receptor agonist, which may be responsible for its analgesic effect.^6^ Chronic nitrous oxide exposure results in dose-dependent inactivation of vitamin B_12_, a co-factor required for methionine synthesis as well as direct inhibition of the enzyme methionine synthase.^7^ Methionine is required for deoxyribonucleic acid synthesis and maintenance of myelin sheaths. ‘Functional’ vitamin B_12_ deficiency secondary to nitrous oxide abuse has been associated with demyelination within the central and peripheral nervous systems.^8^ Other effects of nitrous oxide-induced dysfunctional B_12_ metabolism include agranulocytosis, megaloblastic anaemia with bone marrow suppression, subacute combined degeneration of the spinal cord and reversible psychosis.^7^ In our case, homocysteine levels were normal, suggesting no functional vitamin B_12_ deficiency, which implied that the visual loss was not secondary to chronic nutritional optic neuropathy.

While neuropathy secondary to nitrous oxide use has been a recognised phenomenon since the 1970s,^9^ there have been no reports of recreational nitrous oxide use-associated raised intracranial pressure and associated visual loss. This is the first case we could identify linking recreational nitrous oxide use to fulminant papilloedema. High dose nitrous oxide induced intracranial pressure (ICP) elevation is a well-known phenomenon in the context of induction of anaesthesia with studies showing a mean increase of up to 27 mmHg. The most likely mechanism for this is thought to be cerebral vasodilation leading to increased intracranial blood volume, with quick reversibility in ICP once nitrous oxide is withdrawn.^10,11^ Inhalation of 50% nitrous oxide has also been shown to increase middle cerebral artery blood flow velocity and to impair cerebral autoregulation in healthy people. It is possible that such blood flow variations and loss of autoregulation in the context of chronic high dose nitrous oxide abuse could have triggered a prolonged episode, as demonstrated here. As nitrous oxide was the likely known initiator of this event, it was deemed reasonable to offer a temporary lumbar drain rather than a longer-term CSF shunt.

The thunderclap headache presentation was unusual for raised ICP.^12^ While some could consider this presentation secondary to the drug effect, nitrous oxide is a vasodilator rather than a vasoconstrictor; therefore, this event was likely not attributed to the reversible cerebral vasoconstriction syndrome. The pseudotumor cerebri syndrome can be primary or secondary, both of which have different risk factors. For example, the main risk factor for primary pseudotumor cerebri syndrome, or idiopathic intracranial hypertension (IIH), is weight gain^13^ and while our case was in the obesity category, we cannot be certain that this did not in some way contribute to the mechanism of the raised ICP. Anaemia is a recognised risk factor for secondary pseudotumor cerebri, with up to 10% of cases with ICP having iron deficiency anaemia.^14^ Reynolds et al.^15^ carried out a systematic review to identify other nutrient deficiencies which may be linked to optic disc swelling – vitamin B_12_ deficiency was found to be linked to optic disc swelling in 11 cases, with two of the 11 cases also meeting the diagnostic criteria for IIH. In all cases, vitamin B_12_ deficiency was a result of poor intake, rather than functional deficiency from a toxic cause. In all cases, anaemia, if present, was not severe, suggesting that vitamin B_12_ deficiency may cause neurological dysfunction independent of haemoglobin levels. Vitamin B_12_ deficiency without anaemia resulting in papilloedema has been demonstrated in other case series as well.^16–18^ The mechanism for this is unclear. Perhaps, methionine deficiency directly results in toxic ischaemic optic neuropathy triggering optic disc swelling as an early feature. Additionally, in the context of pseudotumor cerebri, it is yet to be explored what role nutrition and vitamin B_12_ deficiency may have in causing metabolic derangement contributing to increased CSF secretion. Lastly, homocysteinaemia, which results from accumulation of homocysteine in functional vitamin B_12_ deficiency, and lipoprotein a in conjunction with this, have been associated with venous sinus thrombosis, a known cause of intracranial hypertension. In our case, there was no radiological evidence of venous sinus thrombosis, nor evidence of raised homocysteine levels.

Physicians should also be vigilant to nitrous oxide use as a potential triggering factor for raised ICP and visual loss, additionally querying whether nutritional deficiency or malabsorption of vitamin B_12_ may have a role to play in the clinical presentation. A challenge in this case was that insufficient blood samples were received on admission that would have permitted a diagnosis of vitamin B_12_ deficiency prior to starting replacement therapy. In our case, a combination of lumbar drainage and acetazolamide was adopted as successful mechanisms to manage vision-threatening pseudotumour cerebri syndrome.

## Conclusion

The neurological sequelae of chronic nitrous oxide use are well known, however it is only in recent years that the health implications have gained recognition among public health authorities, healthcare providers, and consumers. It is biologically plausible in the context of known neurotoxic effects of nitrous oxide that there exists a link between chronic nitrous oxide use and the development of severe papilloedema and vision-threatening pseudotumour cerebri as seen in this case. Clinicians should consider whether nitrous oxide abuse may be relevant risk factor in the context of a person who presents with papilloedema.
